# Hand-Object Interaction: From Human Demonstrations to Robot Manipulation

**DOI:** 10.3389/frobt.2021.714023

**Published:** 2021-10-01

**Authors:** Alessandro Carfì, Timothy Patten, Yingyi Kuang, Ali Hammoud, Mohamad Alameh, Elisa Maiettini, Abraham Itzhak Weinberg, Diego Faria, Fulvio Mastrogiovanni, Guillem Alenyà, Lorenzo Natale, Véronique Perdereau, Markus Vincze, Aude Billard

**Affiliations:** ^1^ Department of Informatics, Bioengineering, Robotics and Systems Engineering, University of Genoa, Genoa, Italy; ^2^ Vision for Robotics Laboratory, Institut für Automatisierungs- und Regelungstechnik, Technische Universität Wien, Vienna, Austria; ^3^ Robotics, Vision and Intelligent Systems, College of Engineering and Physical Sciences, Aston University, Birmingham, United Kingdom; ^4^ Institut des Systèmes Intelligents et de Robotique, Sorbonne Université, Paris, France; ^5^ Humanoid Sensing and Perception, Istituto Italiano di Tecnologia, Genoa, Italy; ^6^ Institut de Robòtica i Informàtica Industrial, CSIC-UPC, Barcelona, Spain; ^7^ Learning Algorithms and Systems Laboratory, École Polytechnique Fédérale de Lausanne, Lausanne, Switzerland

**Keywords:** hand-object interaction, learning from demonstration, imitation learning, transfer learning, grasping, manipulation, anthropomorphic hands, data extraction

## Abstract

Human-object interaction is of great relevance for robots to operate in human environments. However, state-of-the-art robotic hands are far from replicating humans skills. It is, therefore, essential to study how humans use their hands to develop similar robotic capabilities. This article presents a deep dive into hand-object interaction and human demonstrations, highlighting the main challenges in this research area and suggesting desirable future developments. To this extent, the article presents a general definition of the hand-object interaction problem together with a concise review for each of the main subproblems involved, namely: sensing, perception, and learning. Furthermore, the article discusses the interplay between these subproblems and describes how their interaction in learning from demonstration contributes to the success of robot manipulation. In this way, the article provides a broad overview of the interdisciplinary approaches necessary for a robotic system to learn new manipulation skills by observing human behavior in the real world.

## 1 Introduction

Humans use hands to interact with the environment in everyday activities, e.g., object manipulation, tool usage, deictic gestures, or communication *via* sign language (Napier et al., 1993). The capabilities exhibited by human hands result from a lifetime of learning, observing others, and trying to interact with objects. These abilities enabled us to excel in manipulation tasks, learning new skills, and adapting to complex environments (Lockman and McHale, 1989; Adolph and Franchak, 2017). Robots should dexterously, robustly, and safely manipulate objects to operate in humans’ environments. For example, robots should use tools; or synchronize their movements with humans, either for turn-taking or joint work (Aleotti et al., 2012). However, current robotic hands are unable to match human dexterity. Often state-of-the-art solutions to develop hand-object interaction skills employ learning from human demonstrations to alleviate the need for reliable objects and contact dynamics models (Billard and Kragic, 2019). This approach also allows designing more natural human-like motions, which helps people better understand a robot’s intentions during human-robot interaction (Fukuda et al., 2001). This article is a follow-up to the *Workshop on Hand-Object Interaction: From Human Demonstrations to Robot Manipulation* (HOBI 2020)^1^ at the *IEEE International Symposium on Robot and Human Interactive Communication* held online on September 7, 2020^2^. HOBI 2020 aimed to gather experiences from different fields to discuss the bests conceptual and engineering tools for robots to learn hand-object interaction skills from human demonstration. In this article, the HOBI 2020 organizers and speakers reflect on the open problems and challenges of the aforementioned theme. In particular, this article presents opinions and outlines directions for new research on data acquisition, sensing capabilities, and learning algorithms in the context of transferring human demonstrations of object manipulation to robot platforms. While hand-object interaction has broad interpretations, this article primarily addresses the problem from a functional perspective. More semantically focused aspects, such as social communication, are highly relevant and compatible with the building blocks we present here but necessitate further considerations. The remainder of this article is structured as follows. We define hand-object interaction in Section 2. Opinions and ideas about data acquisition and sensing technologies follow in Section 3. In Section 4, perception algorithms are discussed. In Section 5, we present learning strategies. Finally, Section 6 concludes the article with a discussion on challenges and future work.

## 2 Definition of Hand-Object Interaction

Hand-object interaction has been the subject of different studies in human motor control and robotics (Kang and Ikeuchi, 1995; Stollenwerk et al., 2016). Usually, the state-of-the-art describes the interaction using three main phases: reach, grasp, and manipulation. Although these concepts are intuitive, grasp and manipulation can be confused. Therefore we provide their definition. In particular, Feix et al. (2016) define a *grasp* as:

“every static hand posture with which an object can be held securely with one hand, irrespective of the hand orientation.”

**FIGURE 1:**
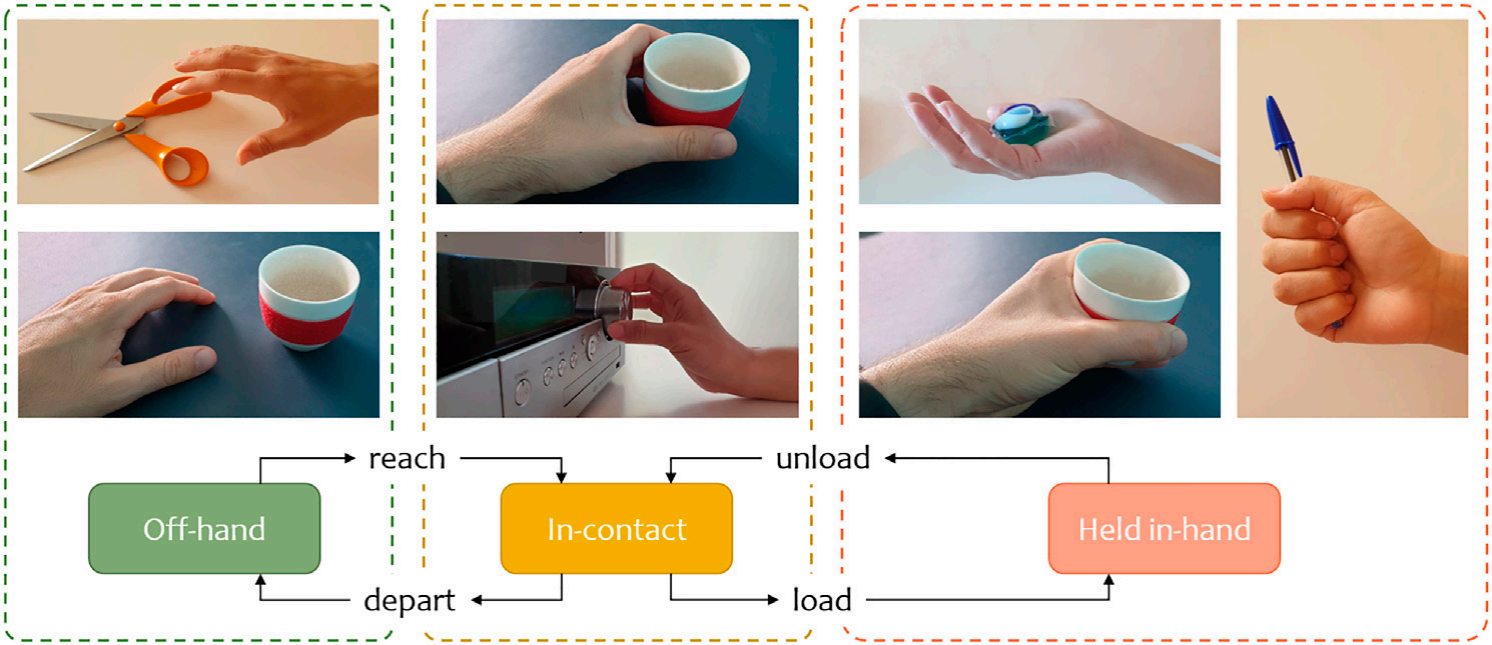
Representation from the object perspective of the hand-object interaction states and transitions.

Instead, to manipulate means to control, use or change something with skill^3^. Formally speaking, we define manipulation as:

“the action changing the state of an object”.

where the object state includes its pose in space and its internal degrees of freedom (DOF), if any. Given this definition, it is clear that grasping is a precondition for manipulation. However, simply referring to these concepts is not sufficient to describe the hand-object interaction and its declinations. Therefore, we divide the hand-object interaction into three states: *Off-hand*, *In-contact*, and *Held in-hand*, see Figure 1.

In the *Off-hand* state, there is no physical interaction between hand and object. However, the hand motion can convey the interaction intention to an external observer. When the hand arrives in the object proximity, the hand-object interaction transitions to the *In-contact* state. Here, the hand can grasp the object and perform limited manipulations since it does not support its weight. When the hand loads the object, the interaction transitions to the *Held in-hand* state. In this state, the human has complete control over the object state to perform free manipulation. The concepts considered in this definition are general. Therefore, this can describe in-hand manipulations purely functional, such as using a tool, or with social intent, such as teaching a manipulation task to an observer.

The described hand-object interaction process is simple and can be adapted to represent bi-manual hand-object interactions as well. It is necessary to point out that this is a high-level description of the hand-object interaction, and we do not intend it as a formal model. For this reason, some aspects, such as hand coordination in bi-manual interaction, are ignored. As previously mentioned, the object state includes both the object pose and the internal DOF. The internal DOF definition is straightforward for rigid objects, e.g., a camera tripod, pen, or scissors. However, for non-rigid objects, the representation is more complex. Deformable objects, such as textiles and foams, are an example since they deform and adopt the shape of the grasping configuration. A recent proposal is to characterize shapeless object grasps in terms of *geometric virtual fingers*, that is, the parameters of the contact surface patch between the finger and the textile and its geometry, that in general reduce to a point, line, and plane (Borràs et al., 2020). Contrary to well-established grasping taxonomies (Cutkosky, 1989; Feix et al., 2016), the object shape cannot influence the definition of the grasp, and external appliances, such as a table, play an essential role in grasping and manipulation enlarging the gripper functionalities. Furthermore, textile manipulation is primarily bi-manual.

## 3 Data and Sensing

Human ability to interact with objects results from the hand’s complex kinematic structure and unparalleled sensing capabilities. Humans, while manipulating objects, use various senses, in particular proprioception and touch. Proprioception is the sense of self-movement and body position that provides continuous feedback. Touch sensing is generated by different mechanoreceptors at different depth levels inside the skin, with higher density in hand and fingertip areas (Vallbo and Johansson, 1984), coming into play when the hand and an object are in contact. The integration of movements with tactile sensing is fundamental, and it is named active touch or haptic perception (Prescott et al., 2011; Seminara et al., 2019). Haptic perception allows humans to perceive objects’ details, optimize grasp stability, and identify shapes and textures. Furthermore, vision helps humans interact with objects, determining object characteristics such as its pose or status (Schettino et al., 2003).

Ideally, an intelligent system needs the same information that humans perceive to learn and perform complex hand-object interactions. This objective justifies the need for multi-modal datasets of human hand-object interactions and the study of advanced robotic sensing capabilities. Such a dataset should clearly describe the hand and object statuses and their interaction. The parameters to describe the hand status can be easy to identify. Hands have a complex kinematic chain for which many models exist. The more detailed ones use 24 DOF to describe the hand joints state, and the hand reference frame is in the palm or wrist center (Romero et al., 2017; Ahmad et al., 2019). Instead, for the object status, providing a unique description is difficult. The description of an object varies according to its characteristics. Given an appropriate definition of a reference frame, the pose of a rigid object consists of 6 DOF. Furthermore, an additional DOF is necessary for each internal articulation, e.g., the DOF of a pair of scissors or a retractable pen.

Describing the interaction between hands and deformable objects using DOF is complex. Not only is the description too high-dimensional to be practical (e.g., any grasping action of a textile would alter its shape), but the type of data observed for the object is almost impossible to obtain through sensorization. State-of-the-art solutions, to handle this complexity, describe garments lying on a table using a polygonal shape (Doumanoglou et al., 2016), singular patches like corners or wrinkles (Kapusta et al., 2019), or cloth parts like collars and hemlines (Ramisa et al., 2016). Other approaches model the interaction focusing primarily on hand trajectories and grasping points (Corona et al., 2018; Zhang and Demiris, 2020). Therefore, the hand-object interaction description necessitates the hand pose and status over time and a reference (often as an image) of the desired object shape.

The information provided by a hand-object interaction demonstration is limited and depends on the used sensing equipment. Vision-based systems, e.g., RGB/RGB-D cameras or Motion Capture environments, can be used to collect information from both hands and objects. However, during the interaction, the hand-object contact creates occlusions, compromising vision-based sensing accuracy. Data from human demonstrations can also be collected instrumenting both hands and objects. To sense hand motions and contacts with objects, data gloves with fiber optic transducers, flex sensors, and inertial measurement units (IMU), as well as force and touch sensors, are often used (Xue et al., 2018; Rashid and Hasan, 2019). Note that hand instrumentation may influence human movements. Similarly, instrumented objects equipped with IMUs and tactile sensors can monitor the object’s status.

A robotic hand, to interact with unknown objects, needs advanced sensing capabilities. These capabilities are helpful both to collect hand-object interaction demonstrations through teleoperation and as feedback for a robot executing an object interaction task. The robot should understand the scene in which it operates, recognize the object’s state, and use, accordingly, the best sensors. In the *Off-hand* state, the robot needs visual perception and proprioceptive information to drive the arm and fingers to reach the object. While grasping, the robotic hand should position the fingertips properly on the object’s surface and distribute forces appropriately. If the object is unknown, different sensing modalities are needed to estimate explicit (e.g., geometry) and implicit (e.g., affordance, grasping properties, and handling possibilities) object properties. When the object is *In-contact* or *Held in-hand*, occlusions prevent external sensors, such as cameras, to provide the robotic system with the needed information. Thus, the system must obtain information through sensors integrated with the fingertips, such as pressure profile sensing arrays, force-torque sensors, or dynamic tactile sensors (Kappassov et al., 2015). Therefore, the development of tactile skins for robotic hands is essential to support object recognition and exploration, improved grasp stability, and more dexterous in-hand manipulation.

## 4 Perception

Perception is the process of interpreting sensory data to represent and understand sensory information. In the learning from human demonstrations context, perception builds the bridge between the sensory input and robot execution. In other words, it derives a mapping between human and robot motions. As discussed in Section 3, different sensors can be used on humans, robots, or in the environment to capture the data.

When perceiving a human hand-object interaction, the objective is to meaningfully explain it, which comprises the spatio-temporal description of the two interacting bodies. This low-level representation can be exploited to understand interaction semantics such as grasp transitions, interaction states, force states, or even task-related modes, thus reaching a high-level interpretation of the events in the scene. For example, detecting the affordance of an object or knowing the previous state of the interaction allows one to reason about possible future actions.

Popular solutions for hand-object interaction perception rely on computer vision techniques (Armagan et al., 2020) to determine the object and hand positions (bounding box or pixel-level location and 3D pose). Moreover, through vision-based approaches, it is possible to extract high-level semantics to improve the understanding of complex interactions. For example, the class of an object allows category-specific information (e.g., stiffness, deformability, weight, etc.) to be retrieved, which may be necessary to adapt the grasp during manipulation.

During hand-object interaction, vision systems encounter numerous difficulties. The object and hand occlude each other such that only a portion of the scene is visible. Moreover, the visual system accuracy can be poor if the grasped object is deformed or presented to the camera under a previously unseen view pose. A possibility to address this issue is to design the learning model to handle multiple components of the occluded objects, e.g., Peng et al. (2020). Another option is to exploit existing Human-Robot Interaction pipelines for automatic image annotation of handheld objects for the object detection task (Maiettini et al., 2017). Finally, refining a pre-trained model on the target hand-object scenario by exploiting unlabeled images from the robot cameras and weakly-supervised learning (Hernández-González et al., 2016; Zhou, 2018) can achieve state-of-the-art accuracy with only a fraction of the required annotated data (Maiettini et al., 2019).

Limitations of vision-based perception can be overcome by increasing the number of sensors. For example, deploying multiple cameras reduces occlusions, enabling more accurate pose estimation, both for the hand and the object (Hampali et al., 2020). Sensorization of objects, human and robotic hands, as introduced in Section 3, is also a viable solution to improve robustness (Rashid and Hasan, 2019). However, these solutions can restrict human hands and may lead to unnatural movement. It is necessary to find a balance between perception richness and human impairment. Furthermore, using different sensing modalities introduces a new challenge, i.e., to merge the data with a coherent perception algorithm. Although some solutions exist to fuse data from various sensor modalities (Li et al., 2020), this problem necessitates further attention. On the other hand, the sensorization of a robotic hand is easier since it is a part of the hardware design process. However, various other challenges arise in perception. For example, motors vibrations and electrical noise can lead to imprecise or even incorrect estimations.

## 5 Learning From Demonstrations

Traditionally, robot manipulation is formalized as a decision-making problem for a Markov Decision Process. In this context, trajectories are discrete, and the actions influence the system state. From a learning perspective, the aim is to optimize a control strategy to perform a sequence of optimal actions to achieve a specific task or a series of related tasks (Plappert et al., 2018). Although huge successes have been achieved for simple hand-object interaction (OpenAI et al., 2019), the optimization formulation is not suitable for all scenarios. In particular, optimal robot behavior may not be easily describable, let alone optimizable. Thus, researchers often turn to expert demonstrations to learn highly advanced and complex skills (Liu et al., 2018; Yu et al., 2018; Smith et al., 2020).

Learning from demonstration allows a robot to learn skills by observing the actions of an expert (Argall et al., 2009; Ravichandar et al., 2020): whether a human (Rajeswaran et al., 2018) or another advanced agent (Hester et al., 2018). A demonstration can be characterized by high-level information (e.g., the state of the object or the manipulator’s joints state) or raw data (e.g., images sequences) (Jain et al., 2019). As an alternative to the direct observation of an expert, teleoperation (i.e., where the exper control either a physical or a simulated robot) is often used to collect data (Zhang et al., 2018), and data gloves are a popular tool to control humanoid robot hands (Rajeswaran et al., 2018). Generate demonstrations by teleoperation avoids the issue of mapping between the human and robot hand kinematics. For this reason, teleoperation is often adopted in challenging scenarios such as the manipulation of clothes (Waymouth et al., 2021).

Data-driven approaches are amongst the most popular for learning from demonstration. While in pure reinforcement learning (RL), the agent continuously interacts with the environment to collect experiences based on the latest policy, in imitation learning (IL), the demonstrations provide the experiences. The expert help reduces the complexity of exploration spaces for learning but introduces other issues such as distribution drift. Therefore, the most successful approaches combine IL and RL by first pretraining a policy with behavior cloning then fine-tuning with policy gradient (Rajeswaran et al., 2018; Radosavovic et al., 2020). Learning from human demonstrations requires adapting the observed motion to the robot kinematics. This problem can be solved by either limiting the analysis to the fingertip poses (Orbik et al., 2021) or considering the full hand motion to preserve the motion naturalness (Meattini et al., 2021). Furthermore, demonstrations are not always optimal, requiring methods to learn from noisy data (Sasaki and Yamashina, 2020). These challenges and others related to learning manipulation from demonstrations are discussed in depth in Zhu and Hu (2018) and Si et al. (2021).

## 6 Conclusion

This article outlined the importance of observing human-object interaction to learn new robotic manipulation skills from demonstrations. To this extent, we provided a general definition of the problem and discussed the interplay between sensing, data acquisition, perception, and learning. We believe several promising research directions are open on the collection and interpretation of data and on learning from it.

When it comes to acquiring demonstrations, an important question remains unanswered: should the data acquisition be non-invasive, for natural interaction, or invasive, maximizing the data richness? The trade-off between invasive and non-invasive sensorization depends on the final task, goal, and algorithm(s) used. A related open question, with implications on the perception and learning pipelines, is what sensing modalities or how many sensors to use. Researchers could be tempted to solve specific challenges by deploying more sensors (e.g., using multiple cameras to cope with occlusion), but this increases the setup costs and complexity, affecting the reproducibility. In similar research fields (e.g., hand and object pose estimation, grasping, and reinforcement learning), the proposal of datasets and benchmarks has favored reproducibility (Hodaň et al., 2018; Armagan et al., 2020; Bottarel et al., 2020; James et al., 2020). However, we observe a lack of standard datasets and benchmarks for complex and dexterous hand-object interaction. Together with the necessity of standardization, the collection of a dataset has to define how to generate optimal demonstrations since state-of-the-art learning algorithms are fragile given noisy input.

Future research should propose standards to simplify data sharing and algorithm evaluation. A suite of shared datasets, evaluation protocols, and metrics will unify the current work enabling more cohesive research. At the same time, to reach human-level manipulation skills, progress is necessary for all the discussed problems. New sensing solutions are needed to increase collected information while preserving a non-invasive setup. Learning algorithms should improve in handling imperfect demonstrations and simplify the adaptation to different kinematics. Furthermore, new hand-object interactions skills should leverage better sensing integration to address challenging scenarios, e.g., manipulation of deformable objects.

## Data Availability

The original contributions presented in the study are included in the article. Further inquiries can be directed to the corresponding author.

## Footnotes

1
https://theengineroom.dibris.unige.it/index.php/hobi/.

2
http://ro-man2020.unina.it/.

3
https://www.oxfordlearnersdictionaries.com/definition/english/manipulate?q=manipulate.
